# Supplementary material for: The Diurnal Blood Metabolome and Effects of Vitamin D Supplementation: A Randomised Crossover Trial in Postmenopausal Women
Source: Int J Mol Sci. 2022 Aug 28;23(17):9748. doi: 10.3390/ijms23179748 (PMC9456020; doi:10.3390/ijms23179748)
Supplement: Supplementary file 1 [file ijms-23-09748-s001.zip › ijms-1840728-supplementary.pdf]

Support Information

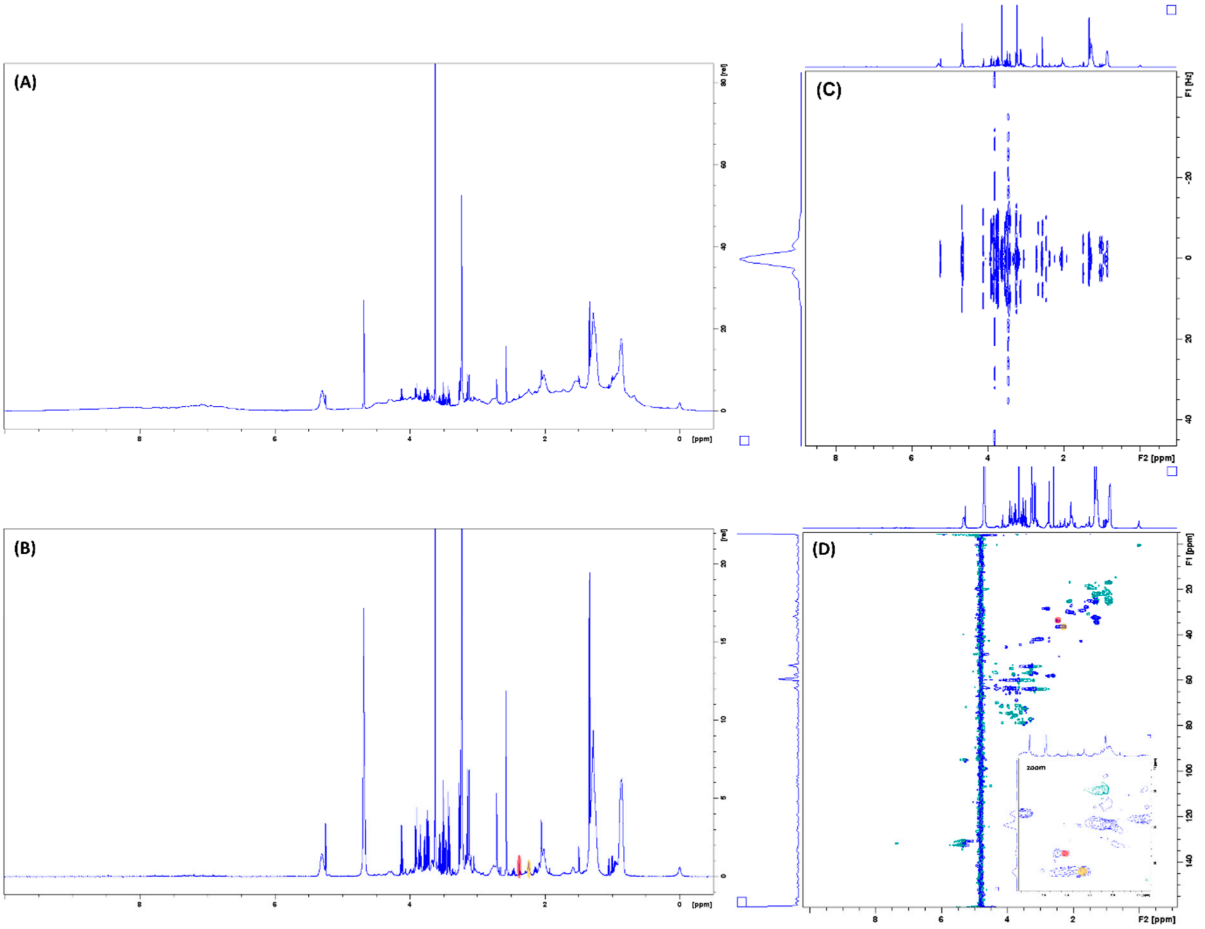

Figure S1. ^{1}H NMR spectrum of a blood plasma sample from a vitamin D supplementated patient (6h after intervention) obtained from A) 1D NOESY presaturation experiment, B) a 1D−Carr−Purcell−Meiboom−Gill (CPMG) spin−echo experiment, C) a 2D − J-resolved experiment, and D) HSQC experiment. Circles in red and yellow show as identification example signals of pyruvate and acetone, respectively.

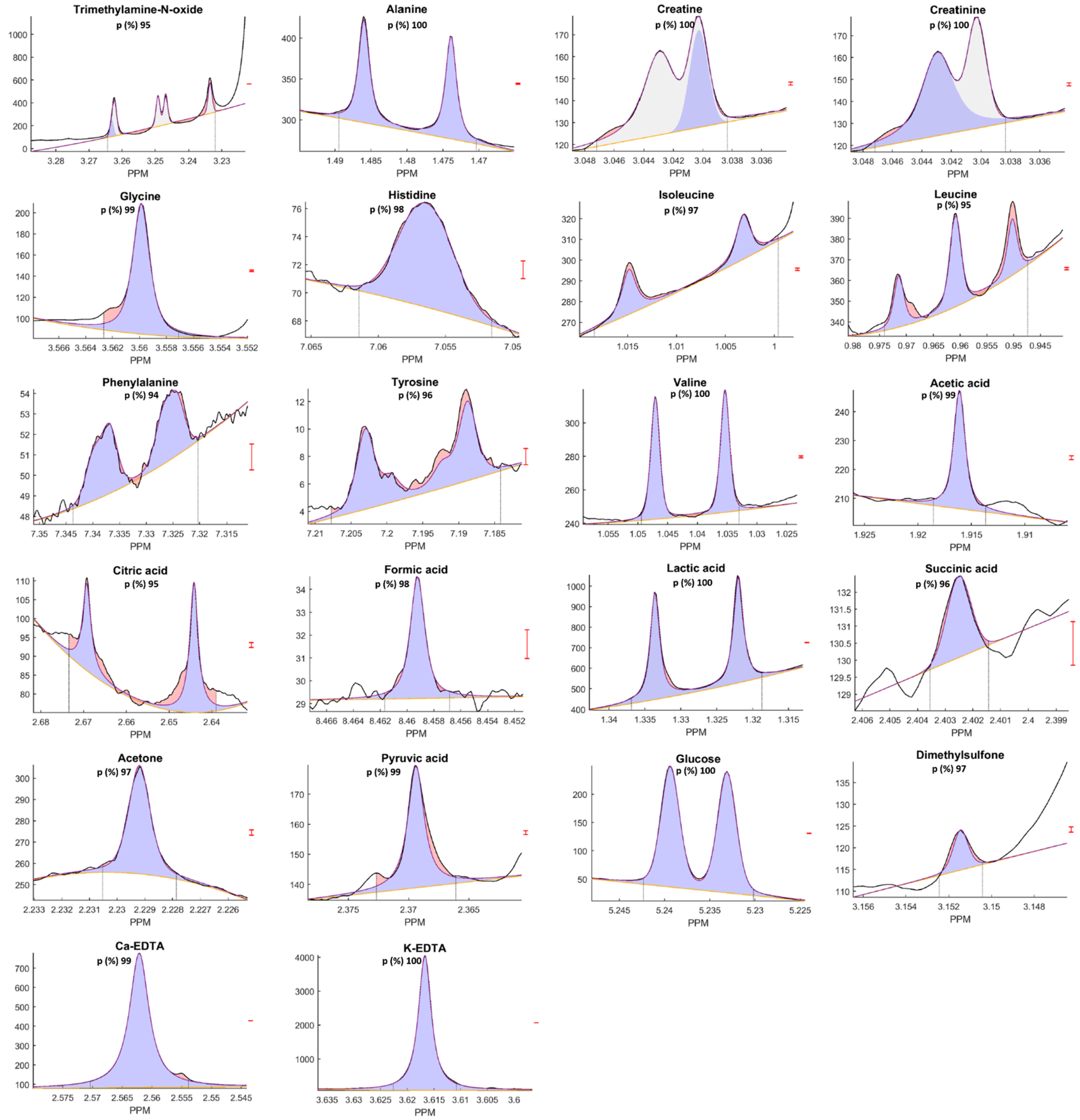

Figure S2: Example of the 22 metabolites signals fitting of a plasma sample with a p-value match above 95%. The fitting was obtained using the Bruker IVDr system. The metabolite average of p-value fitting registering a match above 95% was used as the cutoff of the list of identified metabolites. The black line, the blue line, and the yellow line represent the original spectrum, the calculated signal fit, and its baseline, respectively. The blue area relates to the metabolite concentration to be determined, the red area represents residues, and the grey area presents the sum of all fitted overlapping signals.

Table S1. Concentration in mM (Mean ± CI) of plasma metabolites by time in individuals that received vitamin D supplementation and placebo.

		8AM	10AM	12PM	2PM	4PM	6PM	8PM	8AM/24h		
		**Mean±CI**	**Mean±CI**	**Mean±CI**	**Mean±CI**	**Mean±CI**	**Mean±CI**	**Mean±CI**	**Mean±CI**	**Mean**	**CV %**
**Trimethylamine-N-Oxide**	Vit D	0.00±0.01	0.02±0.02	0.00±0.01	0.02±0.02	0.02±0.02	0.02±0.02	0.02±0.02	0.02±0.02	0.02	40.12
	Placebo	0.00±0.00	0.00±0.01	0.01±0.01	0.02±0.02	0.01±0.01	0.00±0.01	0.01±0.01	0.01±0.01	0.01	55.91
**Alanine**	Vit D	0.38±0.04	0.44±0.03	0.36±0.02	0.45±0.04	0.44±0.04	0.42±0.03	0.48±0.03	0.42±0.04	0.43	8.28
	Placebo	0.39±0.04	0.43±0.03	0.37±0.03	0.41±0.03	0.41±0.03	0.39±0.03	0.47±0.03	0.42±0.04	0.41	7.72
**Creatine**	Vit D	0.03±0.01	0.03±0.01	0.04±0.04	0.04±0.01	0.04±0.01	0.03±0.01	0.05±0.01	0.03±0.01	0.04	22.76
	Placebo	0.03±0.01	0.03±0.01	0.03±0.01	0.04±0.01	0.04±0.01	0.04±0.01	0.06±0.01	0.03±0.01	0.04	27.79
**Creatinine**	Vit D	0.08±0.01	0.08±0.01	0.08±0.01	0.09±0.01	0.08±0.01	0.09±0.00	0.09±0.01	0.09±0.01	0.08	7.12

	Placebo	0.09±0.01	0.08±0.01	0.08±0.01	0.09±0.01	0.09±0.01	0.08±0.01	0.10±0.01	0.09±0.01	0.09	7.76
**Glycine**	Vit D	0.26±0.03	0.26±0.03	0.26±0.03	0.28±0.03	0.26±0.03	0.28±0.03	0.29±0.03	0.29±0.03	0.27	4.72
	Placebo	0.26±0.03	0.25±0.02	0.25±0.03	0.26±0.03	0.25±0.03	0.26±0.03	0.28±0.03	0.29±0.03	0.26	6.12
**Histidine**	Vit D	0.08±0.01	0.07±0.01	0.07±0.01	0.08±0.01	0.07±0.01	0.07±0.01	0.09±0.01	0.08±0.01	0.08	10.86
	Placebo	0.07±0.01	0.06±0.01	0.07±0.01	0.08±0.01	0.07±0.01	0.07±0.01	0.08±0.01	0.07±0.00	0.07	9.52
**Isoleucine**	Vit D	0.05±0.01	0.04±0.00	0.04±0.01	0.07±0.01	0.07±0.01	0.06±0.01	0.08±0.01	0.05±0.01	0.06	24.55
	Placebo	0.05±0.00	0.04±0.01	0.05±0.01	0.07±0.01	0.07±0.01	0.07±0.01	0.08±0.01	0.05±0.01	0.06	23.82
**Leucine**	Vit D	0.07±0.01	0.06±0.01	0.06±0.01	0.10±0.01	0.10±0.02	0.08±0.01	0.11±0.01	0.08±0.01	0.08	21.18
	Placebo	0.07±0.01	0.06±0.01	0.07±0.01	0.09±0.01	0.10±0.02	0.09±0.01	0.11±0.01	0.07±0.01	0.08	21.64
**Phenylalanine**	Vit D	0.01±0.01	0.01±0.01	0.01±0.01	0.03±0.01	0.04±0.01	0.03±0.01	0.04±0.01	0.02±0.01	0.03	43.87

	Placebo	0.02±0.01	0.01±0.01	0.01±0.01	0.03±0.01	0.03±0.01	0.03±0.01	0.04±0.00	0.02±0.01	0.03	42.01
**Tyrosine**	Vit D	0.06±0.01	0.05±0.00	0.05±0.00	0.06±0.01	0.07±0.00	0.06±0.00	0.07±0.00	0.06±0.00	0.06	11.54
	Placebo	0.06±0.00	0.05±0.00	0.05±0.01	0.06±0.00	0.06±0.01	0.06±0.00	0.07±0.00	0.06±0.00	0.06	12.82
**Valine**	Vit D	0.22±0.02	0.19±0.02	0.18±0.01	0.23±0.02	0.22±0.02	0.21±0.02	0.23±0.02	0.21±0.01	0.21	9.33
	Placebo	0.20±0.01	0.19±0.02	0.19±0.02	0.22±0.02	0.21±0.02	0.21±0.02	0.26±0.02	0.21±0.02	0.21	10.79
**Acetic acid**	Vit D	0.03±0.01	0.02±0.00	0.02±0.00	0.03±0.00	0.02±0.01	0.02±0.00	0.03±0.01	0.03±0.00	0.02	12.45
	Placebo	0.03±0.00	0.02±0.00	0.02±0.00	0.03±0.01	0.02±0.01	0.02±0.01	0.03±0.00	0.03±0.00	0.02	18.62
**Citric acid**	Vit D	0.18±0.02	0.20±0.01	0.19±0.01	0.21±0.01	0.19±0.02	0.18±0.02	0.21±0.01	0.18±0.01	0.20	5.62
	Placebo	0.19±0.02	0.20±0.01	0.20±0.01	0.21±0.01	0.20±0.01	0.19±0.02	0.21±0.01	0.20±0.02	0.20	3.61
**Formic acid**	Vit D	0.01±0.01	0.02±0.00	0.01±0.01	0.02±0.00	0.02±0.01	0.02±0.01	0.02±0.01	0.01±0.00	0.02	26.86

	Placebo	0.01±0.00	0.01±0.01	0.01±0.00	0.02±0.00	0.02±0.01	0.02±0.01	0.02±0.01	0.01±0.00	0.02	20.76
**Lactic acid**	Vit D	1.15±0.16	1.58±0.21	1.14±0.13	1.56±0.24	1.40±0.17	1.44±0.21	1.43±0.13	1.29±0.17	1.40	10.77
	Placebo	1.49±0.33	1.56±0.18	1.26±0.15	1.46±0.17	1.41±0.20	1.34±0.15	1.41±0.14	1.42±0.21	1.41	6.74
**Succinic acid**	Vit D	0.00±0.00	0.00±0.00	0.00±0.00	0.00±0.00	0.00±0.00	0.00±0.00	0.00±0.00	0.00±0.00	0.00	116.75
	Placebo	0.00±0.00	0.00±0.00	0.00±0.00	0.00±0.00	0.00±0.00	0.00±0.00	0.00±0.00	0.00±0.00	0.00	61.52
**Acetone**	Vit D	0.01±0.00	0.01±0.00	0.02±0.00	0.02±0.00	0.02±0.00	0.02±0.00	0.02±0.00	0.01±0.00	0.02	28.32
	Placebo	0.02±0.01	0.02±0.01	0.02±0.01	0.03±0.01	0.02±0.01	0.03±0.01	0.03±0.01	0.02±0.01	0.02	22.31
**Pyruvic acid**	Vit D	0.09±0.01	0.11±0.01	0.09±0.01	0.11±0.01	0.11±0.01	0.10±0.01	0.11±0.01	0.09±0.01	0.10	10.73
	Placebo	0.10±0.01	0.11±0.01	0.09±0.01	0.10±0.01	0.10±0.01	0.09±0.01	0.10±0.01	0.09±0.01	0.10	6.84
**Glucose**	Vit D	5.26±0.34	4.79±0.39	4.45±0.17	6.62±0.49	5.74±0.60	5.39±0.42	6.30±0.53	5.40±0.40	5.53	13.99

	Placebo	5.38±0.46	4.89±0.48	4.60±0.25	6.81±0.62	5.92±0.53	5.11±0.37	6.17±0.41	5.56±0.38	5.58	13.99
**Dimethylsulfone**	Vit D	0.00±0.00	0.00±0.00	0.00±0.00	0.00±0.00	0.00±0.00	0.00±0.00	0.00±0.00	0.00±0.00	0.00	30.11
	Placebo	0.00±0.00	0.00±0.00	0.00±0.00	0.00±0.00	0.00±0.00	0.00±0.00	0.00±0.00	0.00±0.00	0.00	12.08
**Ca-EDTA**	Vit D	2.12±0.19	2.21±0.06	2.24±0.03	2.24±0.03	2.17±0.08	2.24±0.03	2.26±0.03	2.22±0.04	2.23	1.28
	Placebo	2.06±0.24	2.20±0.07	2.24±0.07	2.20±0.07	2.19±0.05	2.23±0.06	2.26±0.03	2.28±0.03	2.23	1.55
**K-EDTA**	Vit D	4.68±0.42	5.12±0.39	4.87±0.14	5.26±0.87	5.22±0.83	5.06±0.46	4.97±0.16	5.00±0.19	5.07	2.76
	Placebo	4.68±0.57	4.85±0.24	4.90±0.17	5.04±0.29	4.92±0.26	4.88±0.24	4.86±0.14	5.07±0.22	4.93	1.74

Table S2. Numerical details from the ANOVA and post-hoc analysis of individual plasma according to different time comparing treatments. Comparison column shows the comparisons between different levels that are significant given the p value threshold. Group pairs reported in post-hoc results are given separated by semicolons indicating that the corresponding groups are significantly different

	F-Value	p-Value	-LOG10(p)	FDR	Fischer's LSD
Isoleucine	13.073	3.46E-27	26.462	7.60E-26	Placebo 2PM - Placebo 10AM; Placebo 4PM - Placebo 10AM; Placebo 6PM - Placebo 10AM; Placebo 8PM - Placebo 10AM; Vit D 2PM - Placebo 10AM; Vit D 4PM - Placebo 10AM; Vit D 6PM - Placebo 10AM; Vit D 8PM - Placebo 10AM; Placebo 2PM - Placebo 12PM; Placebo 4PM - Placebo 12PM; Placebo 6PM - Placebo 12PM; Placebo 8PM - Placebo 12PM; Vit D 2PM - Placebo 12PM; Vit D 4PM - Placebo 12PM; Vit D 6PM - Placebo 12PM; Vit D 8PM - Placebo 12PM; Placebo 2PM - Placebo 8AM; Placebo 2PM - Placebo 8AM/24h; Placebo 8PM - Placebo 2PM; Placebo 2PM - Vit D 10AM; Placebo 2PM - Vit D 12PM; Placebo 2PM - Vit D 8AM; Placebo 2PM - Vit D 8AM/24h; Placebo 4PM - Placebo 8AM; Placebo 4PM - Placebo 8AM/24h; Placebo 8PM - Placebo 4PM; Placebo 4PM - Vit D 10AM; Placebo 4PM - Vit D 12PM; Placebo 4PM - Vit D 8AM; Placebo 4PM - Vit D 8AM/24h; Placebo 6PM - Placebo 8AM; Placebo 6PM - Placebo 8AM/24h; Placebo 8PM - Placebo 6PM; Placebo 6PM - Vit D 10AM; Placebo 6PM - Vit D 12PM; Placebo 6PM - Vit D 8AM; Placebo 6PM - Vit D 8AM/24h; Placebo 8PM - Placebo 8AM; Vit D 2PM - Placebo 8AM; Vit D 4PM - Placebo 8AM; Vit D 6PM - Placebo 8AM; Vit D 8PM - Placebo 8AM; Placebo 8PM - Placebo 8AM/24h; Vit D 2PM - Placebo 8AM/24h; Vit D 4PM - Placebo 8AM/24h; Vit D 6PM - Placebo 8AM/24h; Vit D 8PM - Placebo 8AM/24h; Placebo 8PM - Vit D 10AM; Placebo 8PM - Vit D 12PM; Placebo 8PM -

					Vit D 2PM; Placebo 8PM - Vit D 6PM; Placebo 8PM - Vit D 8AM; Placebo 8PM - Vit D 8AM/24h; Vit D 2PM - Vit D 10AM; Vit D 4PM - Vit D 10AM; Vit D 6PM - Vit D 10AM; Vit D 8AM/24h - Vit D 10AM; Vit D 8PM - Vit D 10AM; Vit D 2PM - Vit D 12PM; Vit D 4PM - Vit D 12PM; Vit D 6PM - Vit D 12PM; Vit D 8PM - Vit D 12PM; Vit D 2PM - Vit D 8AM; Vit D 2PM - Vit D 8AM/24h; Vit D 4PM - Vit D 6PM; Vit D 4PM - Vit D 8AM; Vit D 4PM - Vit D 8AM/24h; Vit D 6PM - Vit D 8AM; Vit D 8PM - Vit D 6PM; Vit D 8PM - Vit D 8AM; Vit D 8PM - Vit D 8AM/24h
Phenylalanine	11.484	8.41E-24	23.075	9.25E-23	Placebo 2PM - Placebo 10AM; Placebo 4PM - Placebo 10AM; Placebo 6PM - Placebo 10AM; Placebo 8PM - Placebo 10AM; Vit D 2PM - Placebo 10AM; Vit D 4PM - Placebo 10AM; Vit D 6PM - Placebo 10AM; Vit D 8AM/24h - Placebo 10AM; Vit D 8PM - Placebo 10AM; Placebo 2PM - Placebo 12PM; Placebo 4PM - Placebo 12PM; Placebo 6PM - Placebo 12PM; Placebo 8PM - Placebo 12PM; Vit D 2PM - Placebo 12PM; Vit D 4PM - Placebo 12PM; Vit D 6PM - Placebo 12PM; Vit D 8AM/24h - Placebo 12PM; Vit D 8PM - Placebo 12PM; Placebo 2PM - Placebo 8AM; Placebo 2PM - Placebo 8AM/24h; Placebo 8PM - Placebo 2PM; Placebo 2PM - Vit D 10AM; Placebo 2PM - Vit D 12PM; Placebo 2PM - Vit D 8AM; Placebo 2PM - Vit D 8AM/24h; Placebo 4PM - Placebo 8AM; Placebo 4PM - Placebo 8AM/24h; Placebo 8PM - Placebo 4PM; Placebo 4PM - Vit D 10AM; Placebo 4PM - Vit D 12PM; Placebo 4PM - Vit D 8AM; Placebo 4PM - Vit D 8AM/24h; Placebo 6PM - Placebo 8AM; Placebo 6PM - Placebo 8AM/24h; Placebo 8PM - Placebo 6PM; Placebo 6PM - Vit D 10AM; Placebo 6PM - Vit D 12PM; Placebo 6PM - Vit D 8AM; Vit D 8PM - Placebo 6PM; Placebo 8PM - Placebo 8AM; Placebo 8AM - Vit D 10AM; Vit D 2PM - Placebo 8AM; Vit D 4PM - Placebo 8AM; Vit D 6PM - Placebo 8AM; Vit D 8PM - Placebo 8AM; Placebo 8PM - Placebo 8AM/24h; Placebo 8AM/24h - Vit D 10AM; Vit D 2PM - Placebo 8AM/24h; Vit D 4PM - Placebo 8AM/24h; Vit D 6PM - Placebo

					8AM/24h; Vit D 8PM - Placebo 8AM/24h; Placebo 8PM - Vit D 10AM; Placebo 8PM - Vit D 12PM; Placebo 8PM - Vit D 2PM; Placebo 8PM - Vit D 8AM; Placebo 8PM - Vit D 8AM/24h; Vit D 2PM - Vit D 10AM; Vit D 4PM - Vit D 10AM; Vit D 6PM - Vit D 10AM; Vit D 8AM/24h - Vit D 10AM; Vit D 8PM - Vit D 10AM; Vit D 2PM - Vit D 12PM; Vit D 4PM - Vit D 12PM; Vit D 6PM - Vit D 12PM; Vit D 8AM/24h - Vit D 12PM; Vit D 8PM - Vit D 12PM; Vit D 2PM - Vit D 8AM; Vit D 8PM - Vit D 2PM; Vit D 4PM - Vit D 8AM; Vit D 4PM - Vit D 8AM/24h; Vit D 6PM - Vit D 8AM; Vit D 6PM - Vit D 8AM/24h; Vit D 8PM - Vit D 8AM; Vit D 8PM - Vit D 8AM/24h
Glucose	10.22	4.87E-21	20.313	3.57E-20	Placebo 2PM - Placebo 10AM; Placebo 4PM - Placebo 10AM; Placebo 8AM/24h - Placebo 10AM; Placebo 8PM - Placebo 10AM; Vit D 2PM - Placebo 10AM; Vit D 4PM - Placebo 10AM; Vit D 8PM - Placebo 10AM; Placebo 2PM - Placebo 12PM; Placebo 4PM - Placebo 12PM; Placebo 8AM - Placebo 12PM; Placebo 8AM/24h - Placebo 12PM; Placebo 8PM - Placebo 12PM; Vit D 2PM - Placebo 12PM; Vit D 4PM - Placebo 12PM; Vit D 6PM - Placebo 12PM; Vit D 8AM - Placebo 12PM; Vit D 8AM/24h - Placebo 12PM; Vit D 8PM - Placebo 12PM; Placebo 2PM - Placebo 4PM; Placebo 2PM - Placebo 6PM; Placebo 2PM - Placebo 8AM; Placebo 2PM - Placebo 8AM/24h; Placebo 2PM - Placebo 8PM; Placebo 2PM - Vit D 10AM; Placebo 2PM - Vit D 12PM; Placebo 2PM - Vit D 4PM; Placebo 2PM - Vit D 6PM; Placebo 2PM - Vit D 8AM; Placebo 2PM - Vit D 8AM/24h; Placebo 4PM - Placebo 6PM; Placebo 4PM - Vit D 10AM; Placebo 4PM - Vit D 12PM; Vit D 2PM - Placebo 4PM; Placebo 4PM - Vit D 8AM; Placebo 8PM - Placebo 6PM; Placebo 6PM - Vit D 12PM; Vit D 2PM - Placebo 6PM; Vit D 4PM - Placebo 6PM; Vit D 8PM - Placebo 6PM; Placebo 8PM - Placebo 8AM; Placebo 8AM - Vit D 10AM; Placebo 8AM - Vit D 12PM; Vit D 2PM - Placebo 8AM; Vit D 8PM - Placebo 8AM; Placebo 8PM - Placebo 8AM/24h; Placebo 8AM/24h - Vit D 10AM; Placebo

					8AM/24h - Vit D 12PM; Vit D 2PM - Placebo 8AM/24h; Vit D 8PM - Placebo 8AM/24h; Placebo 8PM - Vit D 10AM; Placebo 8PM - Vit D 12PM; Placebo 8PM - Vit D 6PM; Placebo 8PM - Vit D 8AM; Placebo 8PM - Vit D 8AM/24h; Vit D 2PM - Vit D 10AM; Vit D 4PM - Vit D 10AM; Vit D 6PM - Vit D 10AM; Vit D 8AM/24h - Vit D 10AM; Vit D 8PM - Vit D 10AM; Vit D 2PM - Vit D 12PM; Vit D 4PM - Vit D 12PM; Vit D 6PM - Vit D 12PM; Vit D 8AM - Vit D 12PM; Vit D 8AM/24h - Vit D 12PM; Vit D 8PM - Vit D 12PM; Vit D 2PM - Vit D 4PM; Vit D 2PM - Vit D 6PM; Vit D 2PM - Vit D 8AM; Vit D 2PM - Vit D 8AM/24h; Vit D 8PM - Vit D 6PM; Vit D 8PM - Vit D 8AM; Vit D 8PM - Vit D 8AM/24h
Tyrosine	8.7562	9.11E-18	17.04	5.01E-17	NPlacebo 2PM - Placebo 10AM; Placebo 4PM - Placebo 10AM; Placebo 6PM - Placebo 10AM; Placebo 8AM - Placebo 10AM; Placebo 8AM/24h - Placebo 10AM; Placebo 8PM - Placebo 10AM; Vit D 2PM - Placebo 10AM; Vit D 4PM - Placebo 10AM; Vit D 6PM - Placebo 10AM; Vit D 8AM - Placebo 10AM; Vit D 8AM/24h - Placebo 10AM; Vit D 8PM - Placebo 10AM; Placebo 2PM - Placebo 12PM; Placebo 4PM - Placebo 12PM; Placebo 6PM - Placebo 12PM; Placebo 8AM - Placebo 12PM; Placebo 8AM/24h - Placebo 12PM; Placebo 8PM - Placebo 12PM; Vit D 2PM - Placebo 12PM; Vit D 4PM - Placebo 12PM; Vit D 6PM - Placebo 12PM; Vit D 8AM - Placebo 12PM; Vit D 8AM/24h - Placebo 12PM; Vit D 8PM - Placebo 12PM; Placebo 8PM - Placebo 2PM; Placebo 2PM - Vit D 10AM; Placebo 2PM - Vit D 12PM; Vit D 4PM - Placebo 2PM; Vit D 8PM - Placebo 2PM; Placebo 8PM - Placebo 4PM; Placebo 4PM - Vit D 10AM; Placebo 4PM - Vit D 12PM; Vit D 4PM - Placebo 4PM; Vit D 8PM - Placebo 4PM; Placebo 8PM - Placebo 6PM; Placebo 6PM - Vit D 10AM; Placebo 6PM - Vit D 12PM; Vit D 4PM - Placebo 6PM; Vit D 8PM - Placebo 6PM; Placebo 8PM - Placebo 8AM; Placebo 8AM - Vit D 10AM; Vit D 4PM - Placebo 8AM; Vit D 6PM - Placebo 8AM; Vit D 8PM - Placebo 8AM; Placebo 8PM - Placebo 8AM/24h; Placebo 8AM/24h - Vit D 10AM;

					Placebo 8AM/24h - Vit D 12PM; Vit D 4PM - Placebo 8AM/24h; Vit D 8PM - Placebo 8AM/24h; Placebo 8PM - Vit D 10AM; Placebo 8PM - Vit D 12PM; Placebo 8PM - Vit D 2PM; Placebo 8PM - Vit D 6PM; Placebo 8PM - Vit D 8AM; Placebo 8PM - Vit D 8AM/24h; Vit D 2PM - Vit D 10AM; Vit D 4PM - Vit D 10AM; Vit D 6PM - Vit D 10AM; Vit D 8AM - Vit D 10AM; Vit D 8AM/24h - Vit D 10AM; Vit D 8PM - Vit D 10AM; Vit D 2PM - Vit D 12PM; Vit D 4PM - Vit D 12PM; Vit D 6PM - Vit D 12PM; Vit D 8AM - Vit D 12PM; Vit D 8AM/24h - Vit D 12PM; Vit D 8PM - Vit D 12PM; Vit D 4PM - Vit D 8AM; Vit D 8PM - Vit D 8AM; Vit D 8PM - Vit D 8AM/24h
Leucine	8.066	3.37E-16	15.473	1.48E-15	Placebo 2PM - Placebo 10AM; Placebo 4PM - Placebo 10AM; Placebo 6PM - Placebo 10AM; Placebo 8PM - Placebo 10AM; Vit D 2PM - Placebo 10AM; Vit D 4PM - Placebo 10AM; Vit D 6PM - Placebo 10AM; Vit D 8AM/24h - Placebo 10AM; Vit D 8PM - Placebo 10AM; Placebo 2PM - Placebo 12PM; Placebo 4PM - Placebo 12PM; Placebo 6PM - Placebo 12PM; Placebo 8PM - Placebo 12PM; Vit D 2PM - Placebo 12PM; Vit D 4PM - Placebo 12PM; Vit D 6PM - Placebo 12PM; Vit D 8PM - Placebo 12PM; Placebo 2PM - Placebo 8AM; Placebo 2PM - Placebo 8AM/24h; Placebo 8PM - Placebo 2PM; Placebo 2PM - Vit D 10AM; Placebo 2PM - Vit D 12PM; Placebo 2PM - Vit D 8AM; Placebo 2PM - Vit D 8AM/24h; Placebo 4PM - Placebo 8AM; Placebo 4PM - Placebo 8AM/24h; Placebo 4PM - Vit D 10AM; Placebo 4PM - Vit D 12PM; Placebo 4PM - Vit D 8AM; Placebo 4PM - Vit D 8AM/24h; Placebo 8PM - Placebo 6PM; Placebo 6PM - Vit D 10AM; Placebo 6PM - Vit D 12PM; Placebo 8PM - Placebo 8AM; Vit D 2PM - Placebo 8AM; Vit D 4PM - Placebo 8AM; Vit D 8PM - Placebo 8AM; Placebo 8PM - Placebo 8AM/24h; Vit D 2PM - Placebo 8AM/24h; Vit D 4PM - Placebo 8AM/24h; Vit D 8PM - Placebo 8AM/24h; Placebo 8PM - Vit D 10AM; Placebo 8PM - Vit D 12PM; Placebo 8PM - Vit D 6PM; Placebo 8PM - Vit D 8AM; Placebo 8PM - Vit D 8AM/24h;

					Vit D 2PM - Vit D 10AM; Vit D 4PM - Vit D 10AM; Vit D 6PM - Vit D 10AM; Vit D 8AM/24h - Vit D 10AM; Vit D 8PM - Vit D 10AM; Vit D 2PM - Vit D 12PM; Vit D 4PM - Vit D 12PM; Vit D 6PM - Vit D 12PM; Vit D 8AM/24h - Vit D 12PM; Vit D 8PM - Vit D 12PM; Vit D 2PM - Vit D 8AM; Vit D 2PM - Vit D 8AM/24h; Vit D 4PM - Vit D 6PM; Vit D 4PM - Vit D 8AM; Vit D 4PM - Vit D 8AM/24h; Vit D 8PM - Vit D 8AM; Vit D 8PM - Vit D 8AM/24h
Valine	4.5771	3.87E-08	7.4128	1.42E-07	Placebo 2PM - Placebo 10AM; Placebo 8PM - Placebo 10AM; Vit D 2PM - Placebo 10AM; Vit D 4PM - Placebo 10AM; Vit D 8PM - Placebo 10AM; Placebo 2PM - Placebo 12PM; Placebo 8PM - Placebo 12PM; Vit D 2PM - Placebo 12PM; Vit D 4PM - Placebo 12PM; Vit D 8PM - Placebo 12PM; Placebo 8PM - Placebo 2PM; Placebo 2PM - Vit D 10AM; Placebo 2PM - Vit D 12PM; Placebo 8PM - Placebo 4PM; Placebo 4PM - Vit D 10AM; Placebo 4PM - Vit D 12PM; Placebo 8PM - Placebo 6PM; Placebo 6PM - Vit D 12PM; Placebo 8PM - Placebo 8AM; Placebo 8AM - Vit D 12PM; Placebo 8PM - Placebo 8AM/24h; Placebo 8AM/24h - Vit D 12PM; Placebo 8PM - Vit D 10AM; Placebo 8PM - Vit D 12PM; Placebo 8PM - Vit D 2PM; Placebo 8PM - Vit D 4PM; Placebo 8PM - Vit D 6PM; Placebo 8PM - Vit D 8AM; Placebo 8PM - Vit D 8AM/24h; **Placebo 8PM - Vit D 8PM**; Vit D 2PM - Vit D 10AM; Vit D 4PM - Vit D 10AM; Vit D 8AM - Vit D 10AM; Vit D 8PM - Vit D 10AM; Vit D 2PM - Vit D 12PM; Vit D 4PM - Vit D 12PM; Vit D 6PM - Vit D 12PM; Vit D 8AM - Vit D 12PM; Vit D 8AM/24h - Vit D 12PM; Vit D 8PM - Vit D 12PM
Histidine	4.4215	8.80E-08	7.0553	2.77E-07	Placebo 2PM - Placebo 10AM; Placebo 6PM - Placebo 10AM; Placebo 8AM/24h - Placebo 10AM; Placebo 8PM - Placebo 10AM; Vit D 12PM - Placebo 10AM; Vit D 2PM - Placebo 10AM; Vit D 4PM - Placebo 10AM; Vit D 6PM - Placebo 10AM; Vit D 8AM - Placebo 10AM; Vit D 8AM/24h - Placebo 10AM; Vit D 8PM - Placebo 10AM; Placebo

					2PM - Placebo 12PM; Placebo 8PM - Placebo 12PM; Vit D 2PM - Placebo 12PM; Vit D 8PM - Placebo 12PM; Placebo 2PM - Vit D 10AM; Vit D 8PM - Placebo 2PM; Placebo 8PM - Placebo 4PM; Vit D 2PM - Placebo 4PM; Vit D 8PM - Placebo 4PM; Placebo 8PM - Placebo 6PM; Vit D 2PM - Placebo 6PM; Vit D 8PM - Placebo 6PM; Placebo 8PM - Placebo 8AM; Vit D 2PM - Placebo 8AM; Vit D 8PM - Placebo 8AM; Placebo 8PM - Placebo 8AM/24h; Vit D 2PM - Placebo 8AM/24h; Vit D 8PM - Placebo 8AM/24h; Placebo 8PM - Vit D 10AM; Placebo 8PM - Vit D 12PM; Placebo 8PM - Vit D 6PM; Vit D 2PM - Vit D 10AM; Vit D 4PM - Vit D 10AM; Vit D 8AM - Vit D 10AM; Vit D 8AM/24h - Vit D 10AM; Vit D 8PM - Vit D 10AM; Vit D 2PM - Vit D 12PM; Vit D 8PM - Vit D 12PM; Vit D 2PM - Vit D 4PM; Vit D 2PM - Vit D 6PM; Vit D 8PM - Vit D 4PM; Vit D 8PM - Vit D 6PM; Vit D 8PM - Vit D 8AM; Vit D 8PM - Vit D 8AM/24h
Alanine	3.9531	1.03E-06	5.9865	2.84E-06	Placebo 10AM - Placebo 12PM; Placebo 10AM - Vit D 12PM; Placebo 2PM - Placebo 12PM; Placebo 8AM/24h - Placebo 12PM; Placebo 8PM - Placebo 12PM; Vit D 10AM - Placebo 12PM; Vit D 2PM - Placebo 12PM; Vit D 4PM - Placebo 12PM; Vit D 6PM - Placebo 12PM; Vit D 8AM/24h - Placebo 12PM; Vit D 8PM - Placebo 12PM; Placebo 8PM - Placebo 2PM; Placebo 2PM - Vit D 12PM; Vit D 8PM - Placebo 2PM; Placebo 8PM - Placebo 4PM; Vit D 8PM - Placebo 4PM; Placebo 8PM - Placebo 6PM; Vit D 10AM - Placebo 6PM; Vit D 2PM - Placebo 6PM; Vit D 4PM - Placebo 6PM; Vit D 8PM - Placebo 6PM; Placebo 8PM - Placebo 8AM; Vit D 10AM - Placebo 8AM; Vit D 2PM - Placebo 8AM; Vit D 4PM - Placebo 8AM; Vit D 8PM - Placebo 8AM; Placebo 8AM/24h - Vit D 12PM; Vit D 8PM - Placebo 8AM/24h; Placebo 8PM - Vit D 12PM; Placebo 8PM - Vit D 6PM; Placebo 8PM - Vit D 8AM; Placebo 8PM - Vit D 8AM/24h; Vit D 10AM - Vit D 12PM; Vit D 10AM - Vit D 8AM; Vit D 2PM - Vit D 12PM; Vit D 4PM - Vit D

					12PM; Vit D 6PM - Vit D 12PM; Vit D 8AM/24h - Vit D 12PM; Vit D 8PM - Vit D 12PM; Vit D 2PM - Vit D 8AM; Vit D 4PM - Vit D 8AM; Vit D 8PM - Vit D 6PM; Vit D 8PM - Vit D 8AM; Vit D 8PM - Vit D 8AM/24h
Pyruvic acid	3.611	6.08E-06	5.2162	1.49E-05	Placebo 10AM - Placebo 12PM; Placebo 10AM - Placebo 6PM; Placebo 10AM - Placebo 8AM/24h; Placebo 10AM - Vit D 12PM; Placebo 10AM - Vit D 8AM; Placebo 10AM - Vit D 8AM/24h; Placebo 2PM - Placebo 12PM; Placebo 8PM - Placebo 12PM; Vit D 10AM - Placebo 12PM; Vit D 2PM - Placebo 12PM; Vit D 4PM - Placebo 12PM; Vit D 8PM - Placebo 12PM; Placebo 2PM - Vit D 12PM; Vit D 2PM - Placebo 4PM; Vit D 8PM - Placebo 4PM; Placebo 8PM - Placebo 6PM; Vit D 10AM - Placebo 6PM; Vit D 2PM - Placebo 6PM; Vit D 4PM - Placebo 6PM; Vit D 8PM - Placebo 6PM; Vit D 2PM - Placebo 8AM; Vit D 8PM - Placebo 8AM; Vit D 10AM - Placebo 8AM/24h; Vit D 2PM - Placebo 8AM/24h; Vit D 4PM - Placebo 8AM/24h; Vit D 8PM - Placebo 8AM/24h; Placebo 8PM - Vit D 12PM; Placebo 8PM - Vit D 8AM/24h; Vit D 10AM - Vit D 12PM; Vit D 10AM - Vit D 8AM; Vit D 10AM - Vit D 8AM/24h; Vit D 2PM - Vit D 12PM; Vit D 4PM - Vit D 12PM; Vit D 8PM - Vit D 12PM; Vit D 2PM - Vit D 6PM; Vit D 2PM - Vit D 8AM; Vit D 2PM - Vit D 8AM/24h; Vit D 4PM - Vit D 6PM; Vit D 4PM - Vit D 8AM; Vit D 4PM - Vit D 8AM/24h; Vit D 8PM - Vit D 6PM; Vit D 8PM - Vit D 8AM; Vit D 8PM - Vit D 8AM/24h
Acetone	3.2269	4.30E-05	4.3662	9.47E-05	Placebo 2PM - Placebo 10AM; Placebo 4PM - Placebo 10AM; Placebo 6PM - Placebo 10AM; Placebo 8PM - Placebo 10AM; Placebo 12PM - Vit D 10AM; Placebo 12PM - Vit D 8AM; Placebo 12PM - Vit D 8AM/24h; Placebo 2PM - Vit D 10AM; Placebo 2PM - Vit D 12PM; Placebo 2PM - Vit D 8AM; Placebo 2PM - Vit D 8AM/24h; Placebo 4PM - Vit D 10AM; Placebo 4PM - Vit D 12PM; Placebo 4PM - Vit D 8AM; Placebo 4PM - Vit D 8AM/24h; Placebo

					6PM - Placebo 8AM; Placebo 6PM - Placebo 8AM/24h; Placebo 6PM - Vit D 10AM; Placebo 6PM - Vit D 12PM; Placebo 6PM - Vit D 2PM; Placebo 6PM - Vit D 4PM; Placebo 6PM - Vit D 6PM; Placebo 6PM - Vit D 8AM; Placebo 6PM - Vit D 8AM/24h; Placebo 6PM - Vit D 8PM; Placebo 8PM - Vit D 10AM; Placebo 8PM - Vit D 12PM; Placebo 8PM - Vit D 8AM; Placebo 8PM - Vit D 8AM/24h; Vit D 2PM - Vit D 10AM; Vit D 4PM - Vit D 10AM; Vit D 6PM - Vit D 10AM; Vit D 8PM - Vit D 10AM
Creatinine	2.662	6.94E-04	3.1588	1.39E-03	Placebo 8AM/24h - Placebo 10AM; Placebo 8PM - Placebo 10AM; Vit D 2PM - Placebo 10AM; Vit D 8PM - Placebo 10AM; Placebo 8AM/24h - Placebo 12PM; Placebo 8PM - Placebo 12PM; Vit D 2PM - Placebo 12PM; Vit D 8PM - Placebo 12PM; Placebo 8PM - Placebo 2PM; Placebo 2PM - Vit D 10AM; Placebo 2PM - Vit D 12PM; Placebo 8PM - Placebo 4PM; Placebo 4PM - Vit D 10AM; Placebo 8PM - Placebo 6PM; Placebo 8PM - Placebo 8AM; Placebo 8AM - Vit D 10AM; Placebo 8AM/24h - Vit D 10AM; Placebo 8AM/24h - Vit D 12PM; Placebo 8PM - Vit D 10AM; Placebo 8PM - Vit D 12PM; Placebo 8PM - Vit D 4PM; Placebo 8PM - Vit D 6PM; Placebo 8PM - Vit D 8AM; Placebo 8PM - Vit D 8AM/24h; Vit D 2PM - Vit D 10AM; Vit D 6PM - Vit D 10AM; Vit D 8AM/24h - Vit D 10AM; Vit D 8PM - Vit D 10AM; Vit D 2PM - Vit D 12PM; Vit D 6PM - Vit D 12PM; Vit D 8AM/24h - Vit D 12PM; Vit D 8PM - Vit D 12PM
Creatine	2.3631	2.82E-03	2.5499	5.17E-03	Placebo 8PM - Placebo 10AM; Placebo 8PM - Placebo 12PM; Vit D 8PM - Placebo 12PM; Placebo 8PM - Placebo 2PM; Placebo 8PM - Placebo 4PM; Placebo 8PM - Placebo 6PM; Placebo 8PM - Placebo 8AM; Vit D 8PM - Placebo 8AM; Placebo 8PM - Placebo 8AM/24h; Vit D 8PM - Placebo 8AM/24h; Placebo 8PM - Vit D 10AM; Placebo 8PM

					- Vit D 12PM; Placebo 8PM - Vit D 2PM; Placebo 8PM - Vit D 4PM; Placebo 8PM - Vit D 6PM; Placebo 8PM - Vit D 8AM; Placebo 8PM - Vit D 8AM/24h; Vit D 8PM - Vit D 10AM; Vit D 8PM - Vit D 6PM; Vit D 8PM - Vit D 8AM; Vit D 8PM - Vit D 8AM/24h
Formic acid	2.2511	4.69E-03	2.329	7.93E-03	Placebo 6PM - Placebo 12PM; Placebo 8PM - Placebo 12PM; Vit D 2PM - Placebo 12PM; Vit D 6PM - Placebo 12PM; Vit D 8PM - Placebo 12PM; Placebo 2PM - Placebo 8AM; Placebo 2PM - Vit D 12PM; Placebo 2PM - Vit D 8AM; Placebo 2PM - Vit D 8AM/24h; Placebo 6PM - Placebo 8AM; Placebo 6PM - Vit D 12PM; Placebo 6PM - Vit D 8AM; Placebo 6PM - Vit D 8AM/24h; Placebo 8PM - Placebo 8AM; Vit D 2PM - Placebo 8AM; Vit D 6PM - Placebo 8AM; Vit D 8PM - Placebo 8AM; Vit D 2PM - Placebo 8AM/24h; Placebo 8PM - Vit D 12PM; Placebo 8PM - Vit D 8AM; Placebo 8PM - Vit D 8AM/24h; Vit D 10AM - Vit D 8AM; Vit D 10AM - Vit D 8AM/24h; Vit D 2PM - Vit D 12PM; Vit D 6PM - Vit D 12PM; Vit D 8PM - Vit D 12PM; Vit D 2PM - Vit D 8AM; Vit D 2PM - Vit D 8AM/24h; Vit D 4PM - Vit D 8AM; Vit D 4PM - Vit D 8AM/24h; Vit D 6PM - Vit D 8AM; Vit D 6PM - Vit D 8AM/24h; Vit D 8PM - Vit D 8AM; Vit D 8PM - Vit D 8AM/24h
Lactic acid	1.9375	1.84E-02	1.736	2.89E-02	Placebo 10AM - Placebo 12PM; Placebo 10AM - Vit D 12PM; Placebo 10AM - Vit D 8AM; Placebo 10AM - Vit D 8AM/24h; Vit D 10AM - Placebo 12PM; Vit D 2PM - Placebo 12PM; Placebo 2PM - Vit D 12PM; Placebo 2PM - Vit D 8AM; Placebo 8AM - Vit D 12PM; Placebo 8AM - Vit D 8AM; Placebo 8AM/24h - Vit D 12PM; Placebo 8PM - Vit D 12PM; Vit D 10AM - Vit D 12PM; Vit D 10AM - Vit D 8AM; Vit D 2PM - Vit D 12PM; Vit D 4PM - Vit D 12PM; Vit D 8PM - Vit D 12PM; Vit D 2PM - Vit D 8AM; Vit D 8PM - Vit D 8AM

Acetic acid	1.8564	2.57E-02	1.59	3.77E-02	Placebo 2PM - Placebo 10AM; Placebo 8AM - Placebo 10AM; Placebo 8AM/24h - Placebo 10AM; Placebo 8PM - Placebo 10AM; Vit D 2PM - Placebo 10AM; Vit D 8PM - Placebo 10AM; Placebo 2PM - Placebo 12PM; Placebo 8AM/24h - Placebo 12PM; Vit D 8PM - Placebo 12PM; Placebo 2PM - Placebo 6PM; Placebo 2PM - Vit D 10AM; Placebo 2PM - Vit D 12PM; Placebo 2PM - Vit D 6PM; Placebo 2PM - Vit D 8AM; Placebo 8AM/24h - Vit D 10AM; Placebo 8AM/24h - Vit D 6PM; Vit D 8PM - Vit D 10AM; Vit D 8PM - Vit D 6PM
